# Investigating Neovascularization in Rat Decellularized Intestine: An *In Vitro* Platform for Studying Angiogenesis

**DOI:** 10.1089/ten.tea.2016.0131

**Published:** 2016-12-01

**Authors:** Lindsey Dew, William R. English, Chuh K. Chong, Sheila MacNeil

**Affiliations:** ^1^ Kroto Research Institute, University of Sheffield, Sheffield, United Kingdom.; ^2^ Department of Oncology and Metabolism, School of Medicine, University of Sheffield, Sheffield, United Kingdom.

**Keywords:** decellularization, recellularization, tissue engineering, regenerative medicine, angiogenesis

## Abstract

One of the main challenges currently faced by tissue engineers is the loss of tissues postimplantation due to delayed neovascularization. Several strategies are under investigation to create vascularized tissue, but none have yet overcome this problem. In this study, we produced a decellularized natural vascular scaffold from rat intestine to use as an *in vitro* platform for neovascularization studies for tissue-engineered constructs. Decellularization resulted in almost complete (97%) removal of nuclei and DNA, while collagen, glycosaminoglycan, and laminin content were preserved. Decellularization did, however, result in the loss of elastin and fibronectin. Some proangiogenic factors were retained, as fragments of decellularized intestine were able to stimulate angiogenesis in the chick chorioallantoic membrane assay. We demonstrated that decellularization left perfusable vascular channels intact, and these could be repopulated with human dermal microvascular endothelial cells. Optimization of reendothelialization of the vascular channels showed that this was improved by continuous perfusion of the vasculature and further improved by infusion of human dermal fibroblasts into the intestinal lumen, from where they invaded into the decellularized tissue. Finally we explored the ability of the perfused cells to form new vessels. In the absence of exogenous angiogenic stimuli, Dll4, a marker of endothelial capillary-tip cell activation during sprouting angiogenesis, was absent, indicating that the reformed vasculature was largely quiescent. However, after addition of vascular endothelial growth factor A, Dll4-positive endothelial cells could be detected, demonstrating that this engineered vascular construct maintained its capacity for neovascularization. In summary, we have demonstrated how a natural xenobiotic vasculature can be used as an *in vitro* model platform to study neovascularization and provide information on factors that are critical for efficient reendothelialization of decellularized tissue.

## Introduction

Tissue-engineered constructs are produced to replace damaged, injured, or missing tissues through combining materials technology and biotechnology.^1,2^ While significant progress has been made over the last 30 years, one of the major obstacles in the field is the difficulty in achieving rapid neovascularization when implanting tissues greater than 2 mm in thickness.^3^ When a tissue-engineered (TE) construct is implanted *in vivo*, the transplanted cells require a supply of oxygen to survive. Oxygen diffusion is often the limiting factor in the ability of cells to survive and as a result, few can tolerate being a distance of >200 μm away from a blood vessel.^4,5^ Unfortunately, complete vascularization of TE constructs upon transplantation can take weeks to occur and during this time, the construct cells will become depleted of oxygen and other nutrients, leading to the starvation of the tissue and failure of the construct.^6^

Blood vessel formation is tightly regulated and relies on the chronologically precise adjustment of vessel growth, maturation, and suppression of endothelial cell (EC) growth—all of which are controlled by a large number of factors that influence each other.^7–9^ To induce vascularization within TE substitutes, these same processes will need to occur and neovascularization takes time to occur. The complexity of these tightly regulated processes explains why this remains one of the major obstacles in the field of tissue engineering at present. To overcome this, our aim is to first understand and then translate the basic principles of angiogenesis to produce a physiologically relevant TE construct fit for use in the clinic.

To study the physiological angiogenic environment, it would be a major advantage to have an *in vitro* model that can be used to explore the range of variables (cell types, proangiogenic stimuli, and perfusion rate) that simulate the natural microenvironment. Decellularization is an established technique for producing scaffolds that retain the architecture of the original tissue, including the vasculature and essential biofactors.^10,11^ We suggest that this is an ideal method to form a perfusable scaffold with intrinsic vasculature that can be repopulated with vascular cells surrounded by extracellular matrix (ECM) components to model angiogenesis.

Accordingly, the aim of this work was to generate an acellular natural matrix from rat intestine, repopulate this with vascular and stromal cells, subject these cells to constant perfusion using a bioreactor system to study the requirements for efficient reendothelialization, and see if the development of neovessels could be induced.

## Materials and Methods

### Organ preparation

All surgical procedures and animal husbandry were carried out in accordance with the UK Home Office guidelines under the Animals (Scientific Procedures) Act 1986 and the local ethics committee. Ten adult DBIX rats were sacrificed by isoflurane inhalation and cervical dislocation. A longitudinal abdominal incision was made to expose the abdominal cavity. The inferior vena cava was cannulated using a 24G cannula (Terumo Medical Products, NJ) and flushed with ∼20 mL of heparin (100 U/mL) in PBS to prevent blood clot formation.

An 8–10 cm long segment of the jejunum of the small intestine was isolated and vessels at the peripheries of the segment were ligated before the section of the small intestine was explanted. The intestinal lumen of the isolated segment was then immediately flushed with PBS, 100 U/mL penicillin, 0.1 mg/mL streptomycin, and amphotericin B (0.5 mg/mL). The superior mesenteric artery (SMA) was then cannulated with a 24G cannula under a dissection microscope (M 3Z; Wild Heerbrugg, Germany).

### Decellularization

The cannula in the SMA of the intestine was attached to a peristaltic pump (Watson-Marlow 200 series; Scientific Laboratory Supplies, United Kingdom). dH_2_O was continuously perfused through the vessels at a rate of 2.7 mL/min for ∼3 h with the intestine submerged in dH_2_O. Subsequently, 1% (v/v) Triton X-100 with 0.1% (v/v) ammonium hydroxide in dH_2_O was perfused through the tissue overnight. Finally, dH_2_O was circulated for a further 3 h to remove any residual detergent. The decellularized intestine was then sterilized with 0.1% (v/v) peracetic acid (PA) through the cannula, while submerged in PA for 3 h. This was repeated with sterile PBS thrice to remove any residual acid and then the tissue was stored submerged in PBS at 4°C.

### Histological analyses

Samples were fixed for 24 h at 4°C in 3.7% v/v formalin solution in PBS followed by embedding in paraffin wax and sectioning at 6 μm. Tissue sections were stained with hematoxylin and eosin (H&E) (Sigma-Aldrich), Alcian Blue (Atom Scientific, United Kingdom), PicroSirius Red (Sigma-Aldrich), and modified Verhoeff Van Gieson elastin stain (Accustain; Sigma-Aldrich).

### Vascular patency

After decellularization, 1 mL of blue food dye was injected through the SMA of the intestine to determine the patency of the vessels. The vessels were macroscopically visualized and imaged under a dissection microscope (M 3Z; Wild Heerbrugg). To observe the patency of the vessels microscopically, ∼1 mL FITC-Dextran dissolved in PBS to a final concentration of 100 μg/mL (250 kDa; Sigma-Aldrich) was infused into the intestine and imaged immediately using a Zeiss LSM 510 confocal microscope (Zeiss, United Kingdom).

### DNA quantification

Fresh or decellularized tissue was macerated, lyophilized, and weighed. One hundred milligrams of each sample was digested with proteinase K overnight at 56°C. DNA was then isolated using the DNeasy Blood and Tissue kit (Qiagen, Germany) according to the manufacturer's instructions. The amount of DNA present in each sample was measured by determining the absorbance at 260 nm using a NanoDrop 1000 (Thermo Scientific).

### Collagen quantification

One hundred milligrams of lyophilized tissue was digested in 6 M hydrochloric acid at 121°C overnight and neutralized with 6 M NaOH. Collagen content was measured indirectly by analysis of hydroxyproline content as described previously.^12^ Hydroxyproline concentration was then correlated to collagen content by multiplying by a conversion factor of 5.4 according to Rhaleb *et al.*^13^

### Glycosaminoglycan quantification

Glycosaminoglycan (GAG) content was analyzed using the dimethylmethylene blue (DMB) assay described by Farndale *et al.*^14^ One hundred milligrams of lyophilized tissue was digested with 5 mL of papain digestion solution containing 150 U of papain, 5 mM of l-cysteine hydrochloride, and 5 mM EDTA dissolved in PBS with calcium and magnesium. Samples were incubated for 48 h at 60°C before transferring 40 μL into a clear 96-well plate. DMB solution (250 μL; 16 mg of 1,9 dimethylene blue, 5 mL ethanol, 2 mL formic acid, and 2 g sodium formate adjusted to a volume of 1000 mL using dH_2_O, pH of 3.0) was added to each well and absorbance measured at 520 nm using a microtiter plate spectrophotometer.

### Chick chorioallantoic membrane assay

Fertilized chick eggs (Henry Stewart and Co., Norfolk, United Kingdom) were incubated at a constant temperature of 37°C and humidity of 40%. Seven days postfertilization, a small window was cut into the egg shell. Small segments (∼1 cm in length) of sterilized decellularized intestine or collagen-I gels were placed onto the chick chorioallantoic membrane (CAM). The window was sealed using tape and incubated for 7 days. Changes in the CAM vasculature were imaged through the egg shell window using a USB Digitial Microscope (400 × ; Maplin, United Kingdom). The number of blood vessels <10 μm in diameter growing toward the samples were counted blind by two independent assessors.

### Collagen gel preparation

Working on ice, collagen gels (2 mg/mL) were prepared by adding 0.2 mL rat tail collagen type I (3 mg/mL; Life technologies, Gibco, United Kingdom) to 0.1 mL of (EC) cell growth medium MV. Sterile 1 M sodium hydroxide was added dropwise to neutralize the pH. Supplemented gels were prepared using the same method with the addition of 3 μL of human vascular endothelial growth factor A (VEGFA) (1 ng/μL; Sigma-Aldrich). The gels were then allowed to set in a 37°C incubator for 30 min.

### Recellularization

Vascular structures were reendothelialized using a combination of proliferating human dermal microvascular endothelial cells (HDMECs) derived from juvenile foreskin (Promocell) and human dermal fibroblasts (HDFs), derived as described previously.^15^ Each intestine was flushed with the HDMEC growth medium and allowed to warm in an incubator set at 37°C in a 5% CO_2_ atmosphere before cell seeding. To recellularize with HDMECs alone, 1.5 × 10^6^ HDMECs were resuspended in 1 mL of growth medium and then perfused through the vasculature of the prewarmed decellularized intestine. This was incubated overnight.

To recellularize the intestine with HDMECs and HDFs within the vascular channels, HDMECs were recellularized into the decellularized intestine as described above. This was incubated for at least 3 h. 1.5 × 10^6^ HDFs were then resuspended in 1 mL of HDMEC medium and perfused through the vasculature of the decellularized intestine and incubated overnight. To recellularize the intestine with HDMECs within the vascular channels and HDFs within the intestinal lumen, 0.75 × 10^6^ HDFs were resuspended in 5 mL of HDMEC medium and perfused through the lumen of the intestine. This was incubated (fully submerged in the HDMEC medium) for at least 3 h.

This process was repeated, but this time, the intestine was flipped onto the reverse side so that the HDFs were perfused onto the opposite surface of the lumen. Again, the intestine was incubated for at least 3 h. 1.5 × 10^6^ cells were resuspended in 1 mL of HDMEC growth medium and perfused through the vasculature before incubating overnight. A section of the intestine (from each cell combination) was removed and cultured under static conditions submerged in media, while the rest was attached to a peristaltic pump (Watson-Marlow 200 series; Scientific Laboratory Supplies) through the cannula and a bespoke Lexan polycarbonate bioreactor containing 70 mL of HDMEC growth media.

The samples were placed onto a stainless steel grid to raise them to an air–liquid interface. Gas exchange was maintained with the use of an air filter attached to a port of the container. Perfusion was initially tested at rates of 2.7 mL/min and then 0.5 and 0.025 mL/min to find the influence of the rate of perfusion on endothelialization over 24 and 72 h.

### Production of angiogenesis model

HDMECs were perfused through the vascular channels and HDFs were seeded through the intestinal lumen before placing into the bioreactor as described. Six stainless steel rings were then placed on top of the recellularized intestine. VEGFA collagen gels were prepared as described and 100 μL was pipetted into each metal ring. The bioreactor was then sealed and transferred to an incubator where the gels were allowed to set at 37°C for 15 min. The scaffold was then perfused at 0.025 mL/min for 72 h. Collagen gels without VEGFA were used as controls.

### Immunofluorescence staining of tissue sections

Samples were fixed for 2 h in 4% paraformaldehyde (PFA) w/v in HBSS with Ca^2+^ and Mg^2+^ before dehydrating using graded alcohol washes and embedding in paraffin wax. Sections of 6 μm thickness were cut and mounted on slides. Samples were dewaxed and rehydrated by submerging sequentially in xylene and graded alcohol solutions. Sections were permeabilized using 0.1% (v/v) Triton X-100 for 20 min and incubated in 7.5% (w/v) bovine serum albumin (BSA) at room temperature (RT) for 60 min, followed by washing once with 1% (w/v) BSA in PBS.

Samples were incubated with either polyclonal rabbit anti-laminin primary antibody [1:100 in 1% (w/v) BSA; Abcam, United Kingdom] or polyclonal rabbit anti-fibronectin primary antibody [1:1500 in 1% (w/v) BSA; Abcam] at 4°C overnight. Samples were washed thrice in PBS before incubating with Alexa Fluor™ 488 nm goat anti-rabbit secondary antibody [1:500 in 1% (w/v) BSA; Life Technologies] at RT for 60 min before washing further thrice with PBS. Nuclear counterstaining was performed using DAPI by incubating at RT for 20 min and then washing further thrice with PBS. Samples were imaged using a Zeiss LSM 510 confocal microscope (Zeiss) set at 495 nm λ_ex_–515 nm λ_em_ (FITC) and 358 nm λ_ex_–461 nm λ_em_ (DAPI).

### Immunofluorescence staining of whole-mounted tissue

Samples were fixed in 4% PFA for 20 min at RT. Samples were then quenched with 100 mM glycine, washed once with PBS, and were then blocked for 1 h with 1% BSA (w/v) at RT. Mouse anti-CD31 [1:20 in 1% BSA (w/v); Dako, United Kingdom] and rabbit anti-DLL4 [1:200 in 1% BSA (w/v); Abcam] primary antibodies were added before incubating at RT overnight. The samples were subsequently washed for 2 h in 1% BSA/0.1% Tween 20 (w/v/v/v), followed by incubation with Alexa Fluor 546 goat anti-mouse IgG secondary antibody (Life Technologies) for 2 h at RT. Nuclear counterstaining was performed using DAPI by incubating at RT for 20 min. Finally, the samples were washed with 1% BSA/1% Tween20 for at least 8 h with hourly changes of the BSA/Tween20 solution. Samples were imaged using a Zeiss LSM 510 confocal microscope (Zeiss).

### Live/dead staining

Propidium iodide and Syto 9 (Life Technologies) were diluted in prewarmed DMEM in the ratio 1:3000 under sterile conditions. Sections of intestine were taken, transferred to a six-well plate and incubated at 37°C in 5% CO_2_ for 20 min. Samples were imaged using a Zeiss LSM 510 confocal microscope (Zeiss).

### Statistical analysis

Statistics were performed using a two-tailed, unpaired Student's *t*-test. The significance of the results are denoted by a * symbol (* = *p* < 0.05, ** = *p* < 0.01, *** = *p* < 0.001).

## Results

### Decellularization efficiency

**FIG. 1.**
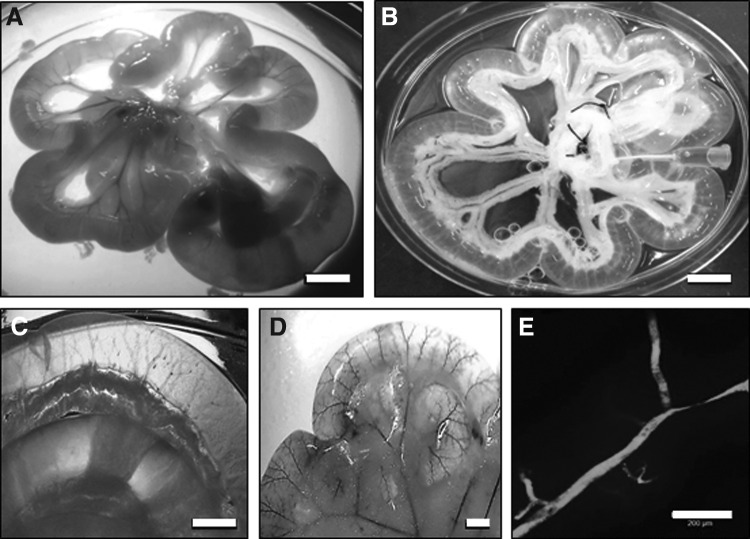
Decellularized intestine maintains vascular patency. Macroscopic view of fresh intestine region of intestine **(A)** and decellularized intestine after treatment with detergent **(B)**. Scale bars are 10 mm. **(C)** Macroscopic view of defined vascular channels remaining after decellularization. Scale bar is 5 mm. **(D)** Evidence of vascular channel patency after the intestine is infused with blue food dye. Scale bar is 5 mm. **(E)** Microscopic evidence of vascular channel patency after infusion with FITC-Dextran. Scale bar is 200 μm.

To decellularize the intestine, the vasculature was used as a transport network to continuously perfuse the nonionic detergent solution containing Triton X-100 and ammonium hydroxide for ∼24 h. The rat intestine became macroscopically transparent after this time and showed good preservation of the mesentery (Fig. 1A, B). The vascular channels of the decellularized intestine were easily seen macroscopically (Fig. 1C). To test the macroscopic patency of the vessels, blue food dye was injected, highlighting the intact vascular structures (Fig. 1D). Injection of FITC-Dextran showed that the microscopic vascular patency was also preserved (Fig. 1E).

### Characterization of nuclei and ECM composition after decellularization

**FIG. 2.**
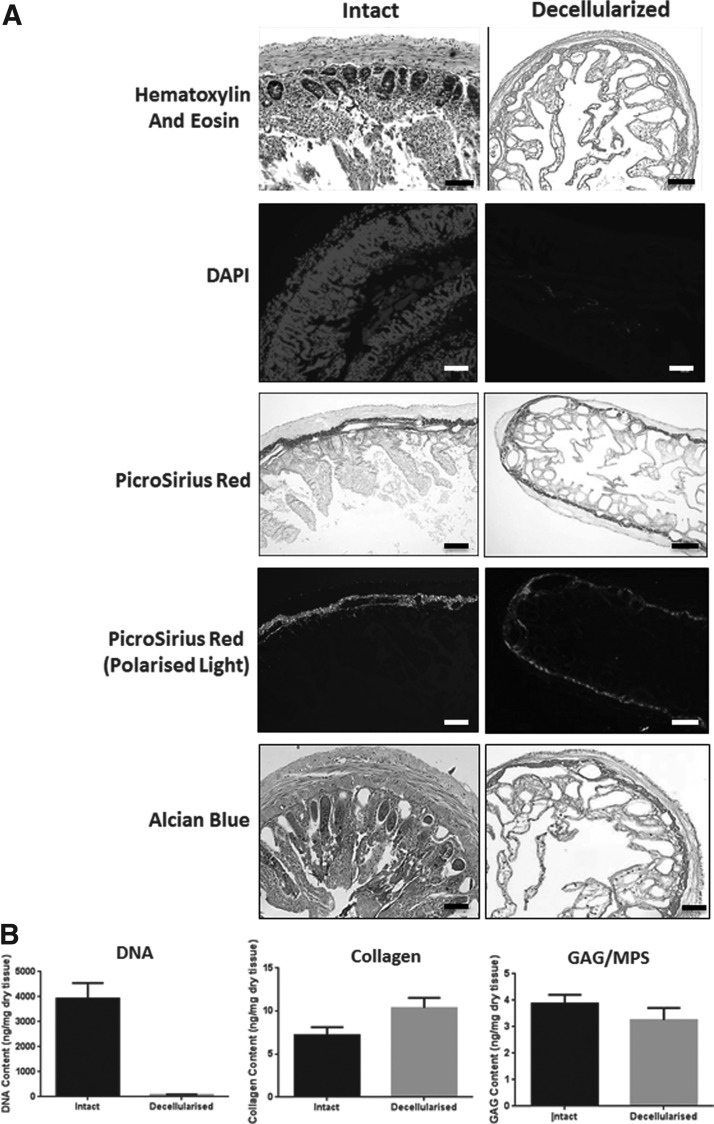
Characterization of the nucleic acid and extracellular matrix content of decellularized intestine. **(A)** Histological staining of nuclei and extracellular matrix content of intact and decellularized intestine. Nuclei were visualized by staining with hematoxylin and eosin or DAPI using light or fluorescence microscopy, respectively. Scale bars are 200 μm. PicroSirius Red staining was used to stain collagen fibers under white light and then imaged under polarized light to detect differences in fibrillar collagen content. GAGs and mucinous polysaccharides (MPS) were detected by staining with Alcian Blue. Scale bars are 200 μm. **(B)** Quantification of DNA, Collagen-I, and GAG content. DNA was quantified after extraction from intact and decellularized intestine by measuring absorbance at 260 nm. Error bars are ± SEM. *N* = 3, *p* < 0.0001. Collagen content was estimated after acid extraction and quantification of 4-hydroxy-proline content. Error bars are ± SEM. *N* = 3 and *p* < 0.05. Quantification of GAG in tissue extracts was performed using the dimethylmethylene blue (DMB) assay. Error bars are ± SEM. *N* = 3, *p* > 0.05. GAGs, glycosaminoglycan.

To characterize the decellularized intestine as a scaffold, the retention of cells and ECM was assessed. H&E staining showed the almost complete removal of cells after the decellularization process, as little to no basophilic staining typical of nuclear material remained (Fig. 2A). This was confirmed on staining of tissue with DAPI. Quantification of DNA after extraction showed that 97% of DNA was removed after the decellularization process (Fig. 2B).

Collagen content was investigated by staining with PicroSirius Red and imaging the samples using brightfield microscopy (Fig. 2A). Collagen is stained red and appears to be retained after decellularization. When examined under polarized light, larger collagen fibers (orange/red) were present, while smaller reticular fibers (green) were largely absent. Quantification of collagen content using the hydroxyproline assay showed a relative increase in content after decellularization in weight-matched samples (Fig. 2B).

**FIG. 3.**
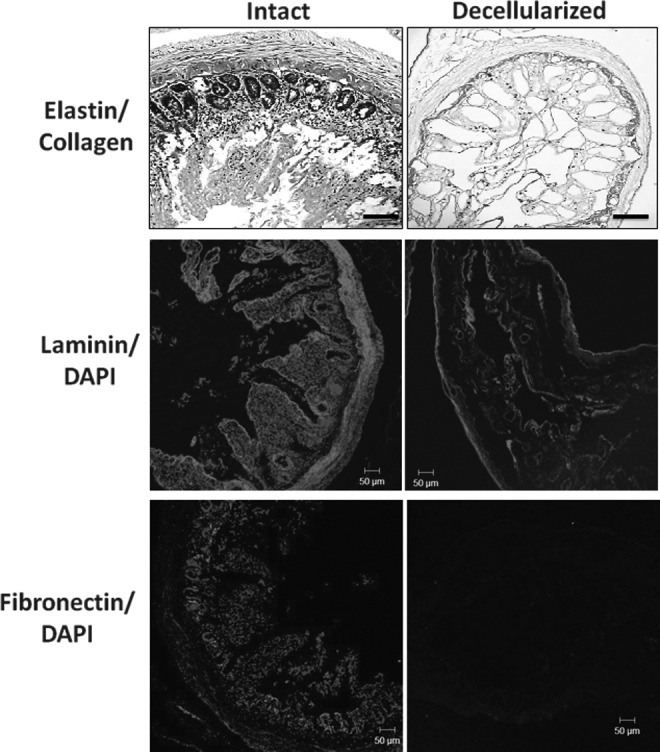
Characterization of elastin, laminin and fibronectin content in intestine before (intact) and after decellularization (decellularized). Verhoeff-Van Gieson staining of FFPE sections of intestine for elastin shown before and after decellularization. Scale bar is 200 μm. Immunofluorescence staining of frozen sections of intestine before and after decellularization for pan laminin (*red*) and counterstained with DAPI (*blue*). Scale bar is 50 μm. Immunofluorescence staining of fibronectin (*red*) and nuclei (DAPI, *blue*) before and after decellularization. Scale bar is 50 μm.

Alcian blue staining of the fresh and decellularized intestine showed little difference in staining of GAGs or mucinous polysaccharides (MPS) (Fig. 2A), and further analysis by GAG quantification showed that there was no significant difference in GAG content after the decellularization process (Fig. 2B). Van Gieson staining showed the almost complete removal of elastin (blue/black) (Fig. 3). Immunofluorescence staining showed the preservation of laminin after the decellularization process, but not fibronectin (Fig. 3).

### Angiogenic properties of the bioscaffold

**FIG. 4.**
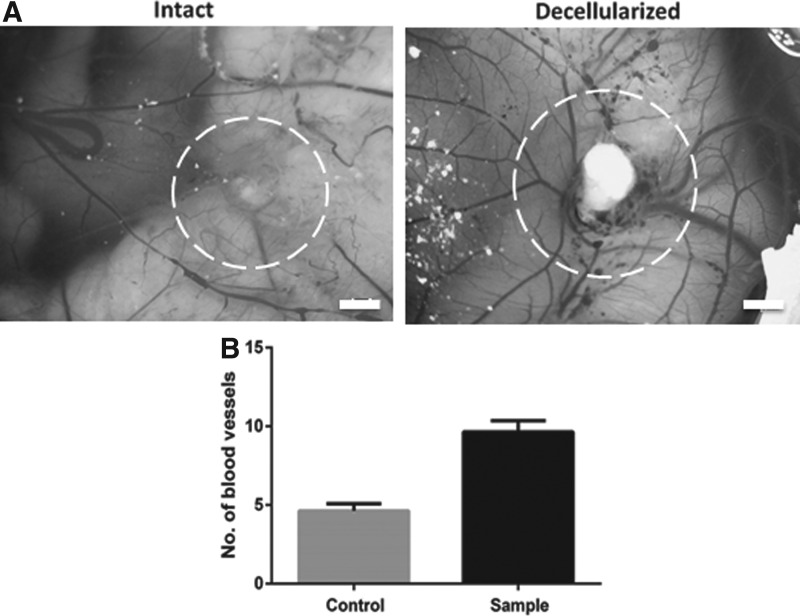
Decellularized intestine retains the capacity to induce neovascularization in the CAM assay. Example photographs of CAM after 7 days postimplantation of a collagen I gel **(A,** negative control) and decellularized intestine **(B)**. The position of the collagen gel and intestine is highlighted by the *dashed circle*. Scale bar is 2 mm. **(C)** Quantification of blood vessels converging toward the implanted material. Error bars are ± SEM. *N* = 3, *p* < 0.0001. CAM, chick chorioallantoic membrane.

To assess the retention of proangiogenic properties of the decellularized scaffold, the CAM assay was used. Sections of decellularized intestine were placed onto chick membranes along with collagen gels as negative controls 7 days postfertilization and incubated for a further 7 days. An increase in vascular density could be seen in the CAM incubated with decellularized intestine (Fig. 4A). Quantification confirmed a significant increase (*p* < 0.0001) in the number of vessels growing toward the decellularized intestine sections compared to the collagen gel control (Fig. 4B).

### The effect of perfusion rates on recellularization with endothelial cells

**FIG. 5.**
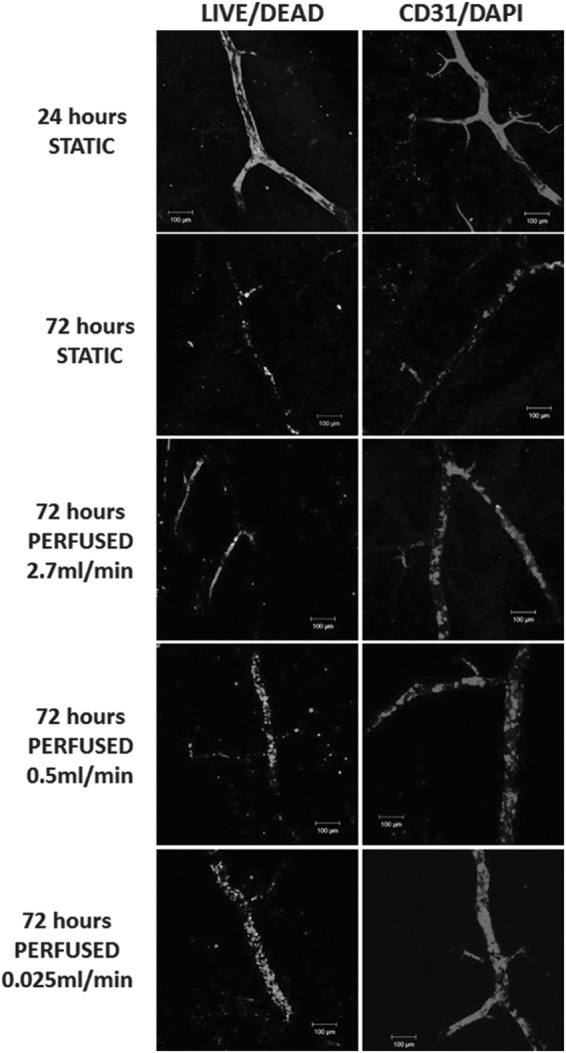
Retention of viable human ECs in the vascular channels of decellularized intestine is dependent on perfusion flow rate. The decellularized intestine was perfused through the vasculature with HDMECs and then cultured for 24 h in the absence of perfusion (24 h STATIC), 72 h without perfusion (72 h STATIC), or for 72 h with perfusion at 2.7, 0.5, or 0.025 mL/min. After the times indicated, the intestine was sectioned and stained to distinguish between LIVE/DEAD cells using propidium iodide and Syto 9 (*left column*). Live cells are shown in *green* and nonviable cells shown in *red*. Alternatively, the intestine was stained to detect HDMECs after fixing at the indicated times using an immunofluorescence whole-mount staining method with anti-CD31 (*red*) and DAPI (nuclei, *blue*). Scale bars are 100 μm. HDMEC, human dermal microvascular endothelial cell.

To assess the ability of the decellularized intestine to act as a scaffold for vessel reconstruction and development of new vessels, HDMECs were infused within the vascular channels and allowed to attach to the matrix. Using a live–dead (green–red) cell fluorescent stain, confocal microscopy showed HDMECs present 24 h after injection with a low proportion of dead cells; however, after 3 days in static culture, few viable cells remained in the vascular channels compared to perfused channels (Fig. 5). To investigate if survival of HDMECs could be improved by perfusion of the vascular channels, HDMECs were initially infused into the scaffolds and cultured under static conditions or perfused at rates of 2.7 mL/min initially and then at 0.5 and 0.025 mL/min (with wall shear stress values of 25.40, 4.70, and 0.24 dyne/cm^2^, respectively).

The choice of an initial perfusion rate of 2.7 mL/min was based on a study of physiological measurements of flow through first-order mesenteric artery branches of rats. This was shown to be 0.3 mL/min.^16^ To determine the flow to be applied through the SMA, the number of branches for each jejunual segment (in this study, 9) was multiplied by this flow rate since the total flow rate through the SMA is equal to the sum of the flow rates through the first-order branches. Using this physical law of continuity, the initial perfusion flow rate through the vasculature was accordingly selected to be 2.7 mL/min.

Further experiments using LIVE/DEAD^®^ analysis and immunostaining for CD31/PECAM-1 (Fig. 5) were then undertaken at much lower flow rates of 0.5 and then 0.025 mL/min. Table 1 shows several experiments in which a qualitative assessment of the extent of endothelialization at two flow rates, 2.7 and 0.025 mL/min, was made.

**Table 1.** Assessment of Extent of Endothelialization in Jejunum with Addition of HDFs and Flow

		*Static*		
*Expt No.*	*+HDFs*	*1 day*	*3 days*	*Perfused (rate mL/min) 3 days*
1	0	+++	ND	ND
2	0	++	+	++ (2.7)
3	0	+++	+	++ (2.7)
4	+HDFs (vessel)	++	+	++ (0.025)
5	+HDFs (lumen)	+++	0	+++ (0.025)
6	0	ND	ND	++ (0.025)
	+HDFs (vessel)	ND	ND	+ (0.025)
	+HDFs (lumen)	ND	ND	+++ (0.025)

The presence of endothelial cells was detected using immunostaining for CD31 and photographs were then scored by three observers blind to the nature of the experiment. Observers were asked to score 0 for no evidence of endothelial cell cover, + for some endothelial cells, but poor (<25% cover), ++ for reasonable, but incomplete endothelial cover, and +++ for complete endothelial cover.

HDFs, human dermal fibroblasts; ND, not done.

These show that, when the lower flow rate of 0.025 mL/min was used, there appeared to be slightly more live cells occupying the vascular channels with a more uniform distribution when compared to the 100 times higher flow rate of 2.7 mL/min. Under static conditions, results were poor. This suggests that HDMECs need some perfusion to survive throughout the vascular network after initial distribution, but the actual rate of flow did not appear critical.

### Recellularization of the decellularized intestine with human vascular cells and fibroblasts

**FIG. 6.**
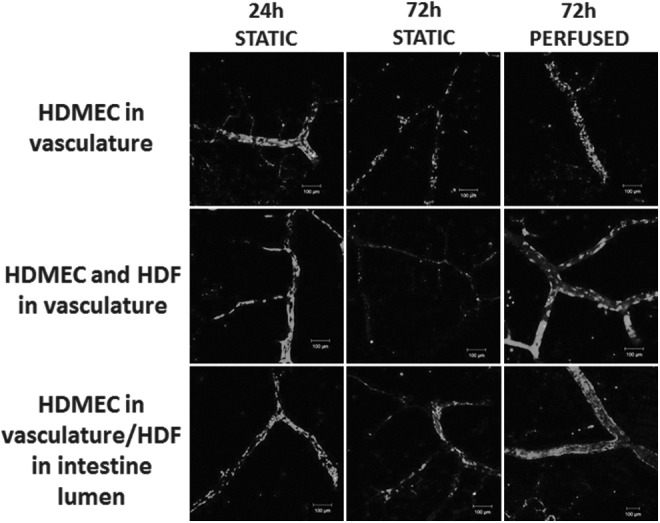
Retention of viable human ECs in the vascular channels of decellularized intestine is increased by continuous perfusion with human fibroblasts. The decellularized intestine was perfused through the vasculature with HDMECs (*top row*) or HDMECs and HDFs (*middle row*), or the vasculature perfused with HDMECs and the intestine lumen perfused with HDFs (*bottom row*). The intestine was then cultured for 24 h in the absence of perfusion (24 h STATIC), 72 h without perfusion (72 h STATIC), or for 72 h with perfusion at 0.025 mL/min. After the times indicated, the intestine was sectioned and stained to distinguish between LIVE/DEAD cells using propidium iodide and Syto 9. Live cells are shown in *green* and nonviable cells shown in *red*. Scale bars are 100 μm. ECs, endothelial cells; HDF, human dermal fibroblast.

As mesenchymal cells such as fibroblasts have been shown to support capillary networks *in vitro* by mimicking pericytes,^7^ HDFs were coinjected into the vascular channels with the HDMECs. Although this did not increase the number of cells present within the vascular channels under static flow conditions, in the presence of flow, a greater number of viable cells could be seen, although cellular coverage of the vasculature did not appear complete (Fig. 6) (also summarized in Table 1). As it is possible that the HDFs are unable to repopulate the precise niche previously occupied by other mesenchymal cells such as pericytes when infused through the vascular channels, HDFs were then infused through the intestinal lumen. This led to greater cellular coverage of the vascular channel lumen with fewer dead cells (Fig. 6).

**FIG. 7.**
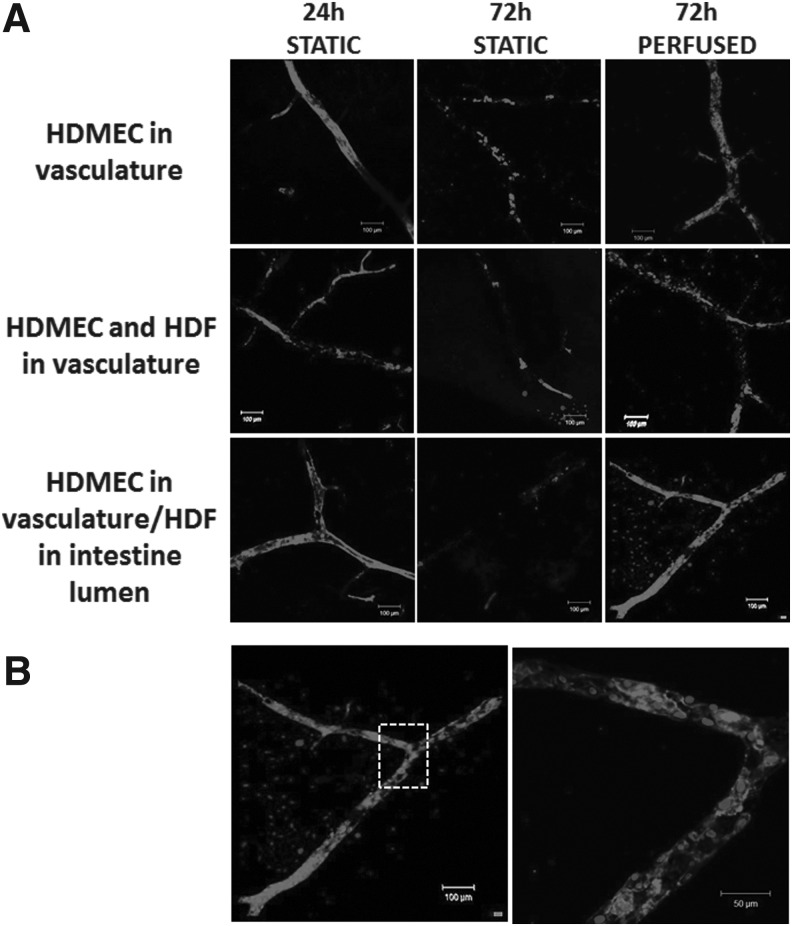
Human ECs form a continuous monolayer within decellularized intestine in the presence of perfusion and human fibroblasts. **(A)** Intestine was repopulated with HDMECs with or without HDFs as described in Fig. 6, before fixing at the times indicated after perfusion of the vascular channels with HDMECs alone, with HDFs in the vascular channels, or with HDMECs in the vascular channels, and HDFs through the intestinal lumen. The intestine was stained after fixing at the indicated times using an immunofluorescence whole-mount staining method with anti-CD31 (*red*) and DAPI (nuclei, *blue*). Scale bars are 100 μm. **(B)** Larger scale images of intestine with HDMECs perfused through the vascular channels and HDFs perfused through the intestinal lumen, after staining for CD31 and nuclei (DAPI, *blue*). Scale bars are 100 and 50 μm.

To further characterize the effects of flow on HDMEC coverage of the vascular channels using the different methods described above, the intestine was stained for the endothelial marker CD31/PECAM-1. As indicated by live–dead staining, HDMEC coverage of the vascular channel after 3 days was significantly improved by the presence of perfusion. In the absence of HDFs, the HDMECs did not form a complete monolayer (Fig. 7A and Table 1). When HDMECs and HDFs were infused through the vasculature, CD31^+VE^ cell staining was decreased, with a large proportion of the cells retained being CD31^−VE^, suggesting that HDFs were lining the vascular lumen (Fig. 7A).

In contrast, when HDFs were infused through the intestinal lumen, CD31^−VE^ nuclei could be seen throughout the tissue in proximity with the vascular channels, suggesting HDF invasion into the decellularized tissue. However, this also led to an increase in coverage of CD31^+VE^ cells within the vascular channels under flow (Fig. 7A). More detailed examination showed that the CD31^+VE^ cells had formed a highly confluent HDMEC monolayer (Fig. 7B).

**FIG. 8.**
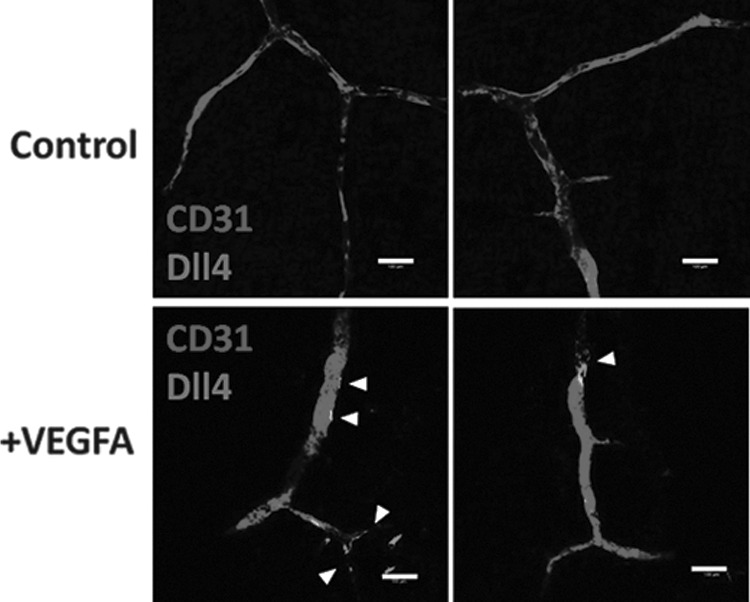
The engineered vasculature within the decellularized intestine maintains the capacity for sprouting angiogenesis. HDMECs were perfused through the vascular channels and HDFs were seeded through the intestinal lumen as described in Figs. 6 and 7 before perfusion at 0.025 mL/min for 72 h. Collagen gels with or without VEGFA were placed on top of the intestine. Intestine sections were fixed and stained for CD31 or Dll4 using immunofluorescence whole-mount staining methods. Example images are shown of intestine with and without VEGFA, and regions positive for Dll4 are indicated by *arrows*. Scale bars are 100 μm. VEGFA, vascular endothelial growth factor A.

We then investigated if the recellularized vascular channels were actively undergoing sprouting angiogenesis by staining for Delta-like ligand 4 (Dll4), a member of the delta–serrate family of ligands that is expressed exclusively in invading capillary tip cells in response to proangiogenic growth factor signaling, including through VEGFA and VEGFR2. Dll4 controls capillary sprout morphology and invasion during sprouting angiogenesis by signaling through Notch in following stalk cells to suppress the tip cell phenotype and is augmented by signaling from stalk cell expression of Jag1 in a highly dynamic process.^17–22^ We were unable to detect Dll4^+VE^/CD31^+VE^ cells in HDMEC vascular channels, indicating sprouting angiogenesis is most likely not active (Fig. 8). However, by placing collagen-I gels loaded with VEGFA on top of the decellularized intestine to generate a proangiogenic growth factor gradient, Dll4 expression could be detected in CD31^+VE^ cells, showing that the recellularized vascular channels retain the capacity for sprouting angiogenesis in the presence of appropriate stimuli (Fig. 8).

## Discussion

Advances in the field of tissue engineering have shown promising steps toward the regeneration of vital tissues and organs, including the skin,^15^ bladder,^23^ urethra,^24^ and trachea.^25^ However, one of the main challenges in almost all cases is the lack of rapid vascularization upon transplantation, which leads to early graft failure. Without an understanding of the factors that affect blood vessel formation and sprouting, it is unlikely that this problem will be solved. Several *in vitro* angiogenesis assays are available, but none combine the ability to include supporting cells (e.g., smooth muscle cells, pericytes or fibroblasts, or other stromal cells), the natural ECM and perfusion, and the introduction of proangiogenic stimuli.^26^

In this study, we aimed to develop an *in vitro* model that will allow all four components to be combined. Continuous perfusion of detergent through the vascular tree of the rat intestine allowed for the production of a natural acellular matrix after ∼18 h. Characterization of the resultant matrix showed a 97% removal of cellular material, while preserving the macro- and ultrastructural components of the native tissue. Dye perfusion showed the preservation of the native vascular network, while histology and quantitative assays showed the preservation of both the GAG and collagen ECM components. The collagen component was shown to increase (relative to other components) after decellularization and is believed to be due to the removal of other components such as cellular proteins. Elastin and fibronectin were both shown to reduce after the process.

This is broadly similar to other reports of decellularization, with some differences in retention of ECM, notably GAG, elastin, and fibronectin, between our studies and those of others.^27,28^ Differences in ECM retention may be due to both the methodology of decellularization and the type of tissues used, and provides an interesting area for further exploration. However, since this study aimed to induce angiogenic sprouting from microvasculature, the loss of elastin was not of major concern since elastin content is important in larger vessels that require elastic recoil, but less important in smaller vessels such as capillaries, which consist of only the tunica intima. As is well established, fibronectin is present both in the serum used in the medium to perfuse the vasculature and is also produced by fibroblasts (e.g., Refs.^29,30^), and was also not considered to be a significant factor in our model.

Results from the CAM assay showed the inherent angiogenic effect of the decellularized intestine. This response argues for the preservation of key growth factors that will ultimately affect cell responses once reseeding takes place. Further characterization is required to identify which proangiogenic stimuli are retained, but this novel finding suggests that decellularized tissue retains some growth factors that can activate angiogenesis in neighboring tissues. This is an important consideration if these decellularized constructs are to be considered for use as a vascular bed to support tissue engineering applications (outside of the remit of this study).

To produce an *in vitro* model to investigate angiogenesis, cells were infused through the vasculature to repopulate the acellular matrix. The response of endothelial cells to shear stress is well known, for example, Ref.^31^ Results clearly showed the necessity for perfusion rather than static culture conditions, but a qualitative analysis (Table 1) showed that perfusion at 0.025 mL/min was only slightly better than 2.7 mL/min for cells to occupy the innate vascular architectures of the scaffold. This suggests that, for this *in vitro* model, there is a wide tolerance to flow as two flow rates varying by 100-fold, both achieved excellent to good endothelialization.

Once the patency of the vascular structures and perfusion conditions was ascertained, the intestine was recellularized with a coculture of HDFs and HDMECs. Studies using mature ECs on their own have shown poor results. Cells have failed to survive and proliferate and most importantly to self-assemble into tube-like structures.^32^ Improved results have been noted by using a coculture of HDMECs and fibroblasts.^33,34^ Cell proliferation and cell signaling have been improved through the use of cocultures, and it is believed that this is as a result of paracrine signaling mechanisms that promote production of VEGF from fibroblasts and the upregulation of VEGF receptors on HDMECs.^32^

The coculture of cells in the decellularized intestinal scaffold was performed and maintained under both static and perfusion cultures. Again, the necessity of perfusion was highlighted. Further investigations using perfusion conditions showed the reorganization of the HDMECs and HDFs over time. After 1 day in culture, results showed the positioning of HDMECs and HDFs within the vascular structures of the decellularized intestine. After 5 days in culture under perfusion conditions, the cells had reorganized themselves and the HDMECs lined the vessels and capillaries.

In the recellularized lung model of Petersen *et al.*, only epithelial and endothelial cells were introduced, and mesenchymal cells that could act as pericytes were absent, which may have contributed to the poorly functioning and leaky vasculature seen in this model.^28^

Dll4 is a well-characterized marker of sprouting angiogenesis that is expressed in endothelial cells that become the leading “tip cell” within quiescent vasculature. Tip cells mediate the invasion of precapillary sprouts into the surrounding stroma in response to a diffusion gradient of proangiogenic growth factors, including VEGFA that activate expression of Dll4, suppress proliferation, and activate the invasive phenotype. Dll4 directly suppresses the invasive tip-cell phenotype in following endothelial “stalk cells” by Notch signaling, thus maintaining the invading capillary sprout architecture.^17–21^.

In this study, we used immunostaining of Dll4 to detect evidence of activation of endothelial tip-cell selection, and thus active sprouting angiogenesis, within the vasculature and capillaries containing HDMECs. Although the decellularized intestine clearly initially contained proangiogenic growth factors that could support angiogenesis in the CAM assay, we could not detect evidence of sprouting angiogenesis in the absence of exogenous VEGFA. These data reinforce the usefulness of our model vascular system for studying angiogenesis in a TE construct and may also prove useful for mechanistic studies of sprouting angiogenesis.

This study shows the ability of these cells to reorganize within this matrix and supports its use as a platform technology, in which to model cell behavior within a physiologically relevant and yet completely controllable environment. One key finding was that there may also be physical barriers to cell integration within decellularized tissues if used for support of vascularization. Endothelial cells could be seeded through the vasculature, but HDFs functioned best when introduced through the organs lumen, in common with approaches optimized for the heart and lung where nonendothelial cells were introduced through the ventricle and alveolar space, respectively.^27,28^ This suggests that the vasculature matrix may present a barrier that limits migration of HDFs into the stroma or perivascular niche of the decellularized tissue.

There are clearly limitations in any *in vitro* study, no matter how sophisticated a three-dimensional (3D) model, as it is unlikely to capture the physiological complexity of the *in vivo* situation.

It is difficult, for example, to relate perfusion *in vitro* to *in vivo*. In this study, we have made no attempt to consider transmural flow. Transmural flow is the perpendicular flow rate into the parenchyma. As blood travels along a vessel, some fluid, interstitial fluid, will travel through to the parenchyma. This is important in delivering nutrients to adjacent cells. We have not attempted to calculate the proportion of flow that continues onto the further vessels, which is incorporated into the transmural flow.

Another major challenge in the future will be to extend this to a more complex model, which includes immune cells.

Other excellent studies of recellularized animal vascular nets from the Mertsching group have shown the patency of such vascular nets repopulated with porcine cells and then implanted in pigs^35^ and of a vascular net repopulated with human cells and implanted in a patient.^36^

In conclusion, this work shows that an acellular matrix that retains the vascular patency and major ECM components of the native rat intestine can be produced by decellularization. Upon recellularization, cells attach well, redistribute themselves thoroughly throughout the vascular network, and show evidence of angiogenic sprouting under perfusion conditions. This model not only has the ability to support cells but it also combines ECM components and the ability to perfuse cells under varying conditions. We suggest that this provides a valuable platform in which angiogenesis can be studied *in vitro*, meeting the growing need for 3D models that can be used to study complex systems, which by definition cannot be readily studied in humans or animals, and to produce *in vitro* models that reduce animal experimentation.
